# Understanding experiences and perceptions of perinatal mental health screening tools among under-served groups: A qualitative study of women from ethnic minority communities in the United Kingdom

**DOI:** 10.1371/journal.pone.0345882

**Published:** 2026-04-01

**Authors:** Ngozika Jane Hemuka, Laura Kudrna, Lina Martino, Jason Copp, Samina Saudagar, Kimberley Maynard

**Affiliations:** 1 Research Ethics, Governance and Integrity, Research Strategy and Services Division, University of Birmingham, United Kingdom; 2 NIHR Health Determinants Research Collaboration (HDRC) Sandwell, Sandwell MBC, Oldbury, United Kingdom; 3 Department of Applied Health Sciences, University of Birmingham, United Kingdom; 4 Centre for Urban Wellbeing, University of Birmingham, United Kingdom; 5 Research Sandwell, Public Health Intelligence Team, Public Health Sandwell, Sandwell MBC, Oldbury, United Kingdom; 6 Barnardo’s Smethwick Family Hub, Sandwell, United Kingdom; 7 Sandwell Family Hubs, Oldbury, Sandwell Council, United Kingdom; The University of Melbourne, AUSTRALIA

## Abstract

**Background:**

Perinatal mental health problems, such as depression and anxiety, are major global public health concerns. Screening for mental health problems in pregnancy supports early detection and treatment, improving outcomes for women and their families. However, mental ill health is not consistently identified in routine maternal practice, particularly among women from ethnic minority communities in high income countries like the United Kingdom. Although research has explored mental health screening tools among majority ethnic populations, there remains a notable lack of evidence regarding the experiences of women from ethnic minority communities. To address this, this study investigated these women’s perceptions of perinatal mental health screening tools used by United Kingdom maternity services.

**Methods:**

A qualitative study of women’s experiences and perceptions of perinatal mental health screening tools. Purposive sampling strategy was employed to recruit women from ethnic minority communities from a local charity to participate in online and face-to-face semi-structured interviews and a focus group discussion. Participants were asked about their understanding of and experience with the Generalized Anxiety Disorder scale (GAD), Edinburgh Postnatal Depression Scale (EPDS) and Whooley questions**.** Data were collected on perceptions of what made it easy or hard to be screened using these tools. Analyses were conducted using a framework analysis approach.

**Results:**

Fifteen women took part in the study (12 in interviews; 3 in a focus group), representing Asian, Black African, Bangladeshi, Black Caribbean, Pakistani, and Afghan ethnic backgrounds. Four organising themes were identified after coding. These were: Mixed experiences and perceptions of perinatal mental health screening tools; Factors shaping women’s perceptions and engagement with perinatal mental health screening tools: barriers and facilitators; Lack of continuity, early support, and clear pathways hinders women’s engagement with mental health screening tools; and Recommendations for improvement.

**Conclusions:**

The detection of mental health problems among women from under-represented communities can be improved by considering the identified themes. Key opportunities include being more explicit and culturally sensitive about mental health, explaining the purpose and benefits of screening tools, its process, and potential outcomes and consequences. It is important to allocate sufficient time, ensure privacy during mental health screening appointments, incorporate open-ended questions and avoid ambiguous words in screening tools. The use of mental health screening tools in the perinatal period needs to be delivered in a supportive context in order to address health and health inequalities.

## 1. Introduction

Maternal mental health is a common public health concern worldwide as women may experience considerable biological, social, and psychological changes in the perinatal period. The perinatal period is defined as the time from conception to one year after birth, and it is a critical period associated with an increased risk of developing mental health issues [1]. Perinatal mental health refers to a woman’s mental health and emotional wellbeing from preconception, through pregnancy and up to one year after delivery [2]. Perinatal mental health issues are significant complications that can arise during the perinatal period, including depression, anxiety disorders, and in severe cases, postpartum psychosis [1]. Perinatal mental health is estimated to affect 1 in 10 women in high- income countries [3] and 1 in 5 women in low-and-middle-income countries (“low-resource setting”) are affected by perinatal depression [4]. In the United Kingdom (UK), perinatal depression and anxiety disorders are estimated to affect between 15%−20% of women who access maternity services [5].

Evidence suggests that poor mental health during pregnancy is associated with several negative maternal health outcomes, including preterm labour and preterm birth [6], poor infant outcomes and cognitive and developmental delays in young children [7]. According to Price et al [8], poor perinatal mental health can negatively impact women’s physical health, their parenting capacity, and children’s health and parent-child bonding. Notably, the 2024 MBRRACE-UK – Saving Lives, Improving Mothers’ report attributed a substantial proportion (34%) of deaths occurring between six weeks and a year after the end of pregnancy to mental health-related causes [9]. Hence, effective screening during the perinatal period is crucial for the early detection and management of mental health symptoms, their severity, and the prevention of associated risk factors [10], as well as for identifying wider support needs and signposting or referring to appropriate services.

Beyond pregnancy itself, there are many known drivers of perinatal mental health problems. A global review of 128 systematic reviews about perinatal depression by Al-Abri et al [11] found that the major correlates were history of mental illness, childcare stress or infant temperament, experiencing stressful life events, lack of social support, maternity blues, marital conflicts, history of abuse, intimate partner violence during pregnancy, gestational diabetes, chronic medical conditions, preeclampsia and pre-term birth, exposure to second hand smoke, sleep disturbance. However, this research did not explore cross-cultural or sub-group differences. This is important because women from ethnic minority groups experience the diagnosis and treatment of perinatal depression differently than other groups. For example, a reanalysis of an existing systematic review of UK evidence by Prady et al [12] found reduced identification and management of perinatal mental health problems for ethnic minority women in the UK with potential explanations including translation and interpretation challenges, healthcare professionals’ views that cultural understandings could impede identifying mental health problems, and the time pressure added by interpretation leading to reduced identification.

In recognition of the overall risk that motherhood poses for mental ill health, international bodies such as the World Health Organization recommends screening for postpartum depression and anxiety using a validated instrument, accompanied by diagnostic and management services for women who screen positive for depression and anxiety [13]. Comparably, in the UK, the National Institute for Health and Care Excellence (NICE) recommends that all health professionals should consider asking two depression identification questions using the Whooley Questions at a woman’s first contact with primary care or her booking visit, and during the early postnatal period (usually at 4–6 weeks and 3–4 months), as part of a general conversation about mental health and wellbeing [5]. It is also proposed to consider asking about anxiety using the two-item generalized anxiety disorder scale (GAD-2), and if a woman responds positively, to use the Edinburgh Postnatal Depression Scale (EPDS) or the Patient Health Questionnaire (PHQ-9) as part of a comprehensive assessment [5]. To facilitate this process, a variety of tools have been developed to screen for symptoms of mental health issues. A recent systematic review of systematic reviews found over 70 different tools validated for use in the perinatal period alone but limited research on tools used to measure anxiety [14]. In this study, we consider one of the most used tools from this review: the Edinburgh Postnatal Depression Scale (EPDS) [15]. We also consider one measure of anxiety due to the need for more research on this aspect of perinatal mental health: Generalized Anxiety Disorder Assessment (GAD-7) [16,17], and the Whooley questions [18] (S1 Appendix).

### 1.1. Women’s views of perinatal mental health screening and support

Despite the importance of screening and early detection of mental health concerns, there are limitations to the existing evidence regarding the perceptions of perinatal mental health screening and support, particularly among ethnic minority populations in the UK [19,20]. It is important to note that for this study, ethnic minority refers to all ethnic groups in the UK except White British, encompassing diverse communities including Asian, Black, Mixed heritage and other ethnic groups. According to the 2021 Census, England and Wales had a total population of 59.6 million, predominantly White British (81.7%), with Asian (9.3%), Black (4.0%), mixed (2.9%), and other ethnic groups (2.1%) [21].

Evidence shows a range of factors associated with how perinatal mental health screening tools are perceived and used. An international systematic review of 20 screening tools for mental disorders among female refugees revealed that most screening tools used were adapted to suit the needs of the populations served [19]. In this review, Donnelly and Leavey [19] concluded that there is a paucity of screening tools for refugee women, particularly those in emergency settings, and cultural factors may not be accounted for in the development of existing screening instruments. This review is limited by its focus on studies conducted among female refugees only, and the articles included in the review were limited to those written in English. Only one study was carried out in the UK, and only two studies addressed maternal mental health conditions, one focusing on postpartum depression and the other on perinatal depression [19], which limits understanding to inform perinatal mental health policies to address social, educational and occupational inclusion for women in different contexts.

Similarly, a global review of perinatal mental health screening in migrant populations by Verschuuren et al [20] found that barriers to screening were cultural appropriateness of screening instruments, stigma, language and communication, and family, while facilitators were interpreters, positive patient-provider relationship, training and education for healthcare providers, and availability of assistance for women during screening. This study was limited by its focus on first generation migrants’ populations only, however, a strength was its comprehensive and rigorous search strategy, which included no language restrictions [20]. There were only three studies with UK samples identified in the review and all were quantitative studies [22–24], which limits an understanding of how ethnic minority women describe the barriers and opportunities for improvement in use of the tools.

More evidence confirms the ongoing trend of disparity in screening and support, showing a range of factors that impact access to perinatal mental health support for ethnic minority women. Another systematic review by Watson et al [25] looked at evidence from Europe, exploring ethnic minority women’s experiences of perinatal mental ill health, help-seeking and perinatal mental health services. The review highlights factors that contribute to more limited access to perinatal mental health support for ethnic minority women, encompassing limited awareness of mental health issues, sociocultural factors such as cultural expectations and stigma, and organisational challenges including culturally insensitive and fragmented health services, culturally incompetent and dismissive attitudes among healthcare providers, which impact the ability of ethnic minority women in the UK to engage with perinatal mental health services [25]. While all 15 studies included in the review were undertaken in the UK, women’s perceptions of screening instruments were not explored [25], which is a gap that the current study addresses.

A further Europe-based systematic review conducted by Waqas et al [26] investigated whether screening improves maternal mental health and infant outcomes, and it additionally extracted information about the perceived acceptability and feasibility of screening programmes. In general, perceptions of screening programmes by providers were positive, and it was reported that they resulted in better attitudes and practices towards treatment-seeking. There were high completion rates and high satisfaction. Key barriers and facilitators to uptake of these screening interventions were about healthcare provider empathy, education, stigma, and listening practices [26]. However, the review results were not disaggregated by ethnicity, and only one UK study contributed to the findings, which was a trial that also did not disaggregate the results by ethnicity [26].

Another systematic review by Webb et al [27] focussed on high-income countries, including seven from the UK, and investigated the general drivers of implementing perinatal mental health care in health and social care settings. There were a wide range of drivers across multiple levels, including individual (such as health beliefs, family), healthcare provider (such as knowledge, training, confidence), interpersonal (such as trusting relationships, language barriers,) organisational (service integration, design and delivery of care), political (policy, funding), and societal factors (stigma, culture) [27]. Again, however, results were not specific to an ethnic minority sample and were about implementation more broadly, not of screening tools in particular.

Smith et al [28] also conducted a systematic review and meta-synthesis of qualitative studies in the UK, and examined the barriers to accessing mental health services for women with perinatal mental illness. Barriers to accessing mental health services for women with perinatal mental illness were identified at four levels, including individual (stigma, poor awareness), organisational (resource inadequacies, service fragmentation), sociocultural (language, cultural barriers) and structural (unclear policy) levels [28]. However, the review results were not specific to women’s perception of assessment tools but focused on barriers to accessing appropriate care.

Previous global reviews also highlight that language barriers and lack of cultural sensitivity among healthcare professionals contribute to reduced access to and engagement with perinatal mental health services among women from minority ethnic backgrounds [29,30]. Nevertheless, both reviews focused exclusively on migrant women and addressed access to prenatal mental health services in general, rather than screening tools specifically [29,30]. A population-based study examined two datasets from the National Commissioning Data Repository to explore access rates to secondary mental health services, including involuntary admissions to psychiatric inpatient care and patterns of engagement for ethnic minority women who had their babies in 2017 in the UK [31]. Findings from the study suggests that women from minority ethnic groups had significantly lower access to community mental health services than their White British counterparts, despite experiencing similar, or higher levels of distress [31]. Indian women had statistically significantly lower admission rates than White British women [31]. Although this study focused on ethnic minority groups, it explored women’s access to perinatal mental health services and not screening tools specifically [31].

Screening approaches can influence individuals’ acceptance of and comfort with mental health screening. A qualitative study assessed if a digital perinatal mental health screening programme is feasible and acceptable for 22 women of refugee and migrant backgrounds in Australia [32]. In this study, Willey at al [32] showed that in general, women have positive experiences of digital perinatal mental health screening programme and found it feasible and acceptable for screening perinatal mental health problems. Nevertheless, this study is limited to refugee and migrant women in Australia [32]. Another qualitative study conducted among 11 White women and four women from diverse ethnic backgrounds in Calgary, indicates that expressing mental health concerns during screening was challenging for several reasons [33]. These include difficulty to recognise and understand symptoms, fear of the consequences of disclosing issues or being judged, lack of continuity in their care and lack of feedback [33]. Accordingly, clarity around the outcomes of disclosing mental health concerns, and the availability of immediate support, could help to dismantle the barriers to communicating mental health issues and encourage honest answers during screening [33]. This study raises important issues; however, it was conducted in a Canadian context that could have different challenges than a UK context due to differences in population demographics and healthcare systems. The present investigation focuses on mothers from minority ethnic backgrounds in the UK.

### 1.2. Summary of limitations of existing evidence

In summary, the limitations of existing review evidence are a focus on migrant [20,29,30] and refugee populations [19], lack of disaggregating the results by ethnicity [26], and a focus on access to healthcare services [25,28], or implementation more broadly rather than the screening tool itself [27]. The existing qualitative evidence is limited by its focus on migrant and refugee populations [32] and by the examination of women in countries other than the UK [32,33]. Addressing these gaps is essential to developing culturally attuned and contextually relevant screening tools that can enhance the acceptability of and engagement with perinatal mental health services in the UK. Further research in this field is required to inform public health policies to address perinatal mental health inclusion for ethnic minority women in different contexts. Thus, this research aimed to explore the experiences and perceptions of perinatal mental health screening tools among women from ethnic minority communities in the UK. This research builds upon the foregoing studies by exploring similar questions but concentrating on a UK context.

## 2. Contribution of research

To the best of our knowledge, we propose the first qualitative study to focus on how women from ethnic minority backgrounds in the UK perceive screening tools for identifying mental health issues during the perinatal periods.

### 2.1. Research questions

Building on existing literature, there were four research questions: (1) What are the experiences and perceptions of perinatal mental health screening tools among women from ethnic minority communities in the UK? (2) What barriers and facilitators do women describe as shaping their perception of the effectiveness of perinatal mental health screening tools in ethnic minority communities? (3) What strategies and recommendations can be proposed to address the identified barriers?

## 3. Methods

This methods section was written in accordance with the Standards for Reporting Qualitative Research (SRQR) reporting checklist for qualitative studies [34].

### 3.1. Patient and Public Involvement and Engagement (PPIE)

Public involvement was embedded into the current study to inform the recruitment of participants and the development of the research aim, questions, and topic guide. This was achieved by hosting a wellbeing engagement event at a local charity (Barnardo’s Family Hub) and meetings with contributors. A total of ten women from various ethnic backgrounds, including African, Indian, and White other ethnicities, all of whom were mothers, provided input; however, it was not known whether they had experienced perinatal mental health difficulties. For example, they provided valuable insights and feedback regarding the topic’s relevance to their communities and the study design. They expressed preference for a qualitative design to enable in-depth exploration and foster social interaction among participants. They also helped shape the research questions, highlighting the need to examine barriers and facilitators in communicating and responding to screening tools. There was no prior relationship between the researchers and the ten women who participated in the PPIE event. While the researchers had not previously worked with the charity in this context, the local government maintains an established partnership with the charity.

### 3.2. Study design

This was a qualitative study using semi-structured interviews and a focus group to provide flexibility in data collection while exploring participants’ personal and sensitive insights and experiences, as well as rich group dynamics through shared perceptions. The use of both methods was therefore well suited to gathering nuanced qualitative data to address the research questions while considering participants’ privacy concerns (for interviews) and the dynamics of group discussions in focus groups [35].

### 3.3. Participants and sampling

Participants were recruited from Barnardo’s Family Hub in Sandwell, UK. Eligible participants met the following inclusion criteria: (1) aged 18 years or older; (2) experience of being screened for mental health concerns during the perinatal period; (3) belonging to an ethnic minority community in the local UK region of Sandwell, West Midlands; and (4) willing to take part in the study. The local UK region of Sandwell, West Midlands was selected because it is a super-diverse area [36,37], and their local government team has a close partnership with Barnardo’s Family Hub who was able to provide a Punjabi interpreter and support purposive sampling participants from under-served groups with diverse experiences (e.g., migration, ethnicity, and women who do not speak English fluently, as they are often excluded from research studies) [38]. Participants were recruited via the distribution of advertising flyers, local website and social media sites, and oral announcements during women’s maternal classes. All participants received a £15 shopping voucher honorarium for taking part and reimbursement for travel expenses. An expected sample size of 10–12 individual participants were initially estimated based on prior research [39]. However, recruitment continued until no new themes were identified, and thematic saturation was achieved [40]. This was the point at which the new interviews were producing little or no new useful information [40].

### 3.4. Data collection

All data collection occurred in June 2024 and was in person at Barnardo’s Family Hub apart from one online interview via Microsoft Teams. Interviews and focus group were conducted by two female interviewers (NJH; an embedded researcher with prior qualitative research experience and an interest in maternal mental health and SS; a staff member from Barnardo’s Family Hub with experience in conducting community consultation interviews for community development initiatives). Interview and focus group discussions were audio-recorded, transcribed verbatim, read and sense-checked against the audio recordings. The interview items were informed by contributions from PPIE members and a comprehensive review of the relevant literature [25,33,41], ensuring alignment with established evidence and key themes. Contributions from PPIE members informed the development process, helping to ensure that the questions were relevant, clear, and appropriately framed for participants.

In brief, this covered experiences of being asked questions about their mental health during pregnancy and after the birth of their babies, their views on the effectiveness of screening tools used during and after pregnancy for identifying mental health problems, and the barriers and facilitators that impact their perceived effectiveness of the mental health screening tools for expectant and new mothers from ethnic minority communities. The semi-structured topic guide is in the Supplemental S2 Appendix. Participants were also asked to comment on their understanding and sensitivity of words on the mental health screening scales and if they felt comfortable answering the questions truthfully.

### 3.5. Researcher reflexivity

Reflexivity was embedded throughout the research process to enhance transparency and critical awareness of how researchers’ positions and experiences might shape the study. The research team actively reflected on their own perspectives and assumptions regarding perinatal mental health, acknowledging that their professional backgrounds, personal experiences, and cultural contexts could influence how data were interpreted, and meaning was constructed. To identify and mitigate potential biases, reflective and reflexive practices were employed during data collection and analysis [42]. The principal investigator (NJH) gave careful consideration to the influence of her position as a female researcher from an ethnic minority background, particularly in relation to how shared or differing experiences with participants might shape interpretations of the data. No prior relationship was established with participants before the commencement of the study, ensuring that data were grounded in participants’ accounts rather than pre-existing rapport. This reflexive approach involved the use of reflective tools, including maintaining a physical research diary to document evolving thoughts, emotional responses, methodological decisions, and shifts in interpretation throughout the study [43]. This ongoing record facilitated transparency and allowed for continual questioning of assumptions as the research progressed. In addition, reflexivity was practiced as a collective endeavour [43]. The principal investigator engaged in regular, deliberate discussions with the co-investigator (LK), a White female researcher from the United States to examine how individual, diverse, and shared perspectives influenced analytical choices and thematic development. These discussions created space for constructive challenge and critical reflection, helping to ensure that interpretations were co-constructed through multiple lenses rather than driven by any single researcher’s standpoint [43].

### 3.6. Data analysis

Transcribed interviews were anonymised and analysed using framework analysis. This is a systematic approach to analysing qualitative data that involves developing and applying a matrix-based analytical framework to identify, describe, and interpret key patterns and themes within a dataset [44]. This data analysis technique was chosen to facilitate a systematic approach (including familiarisation, coding, indexing, and charting) for analysing and interpreting data, ensuring that the insights derived are accessible and valuable for policymaking [45]. All transcripts were read several times to facilitate data familiarisation. Data analysis was initially conducted by (NJH). Findings and themes were refined in triangulation meetings with the co-investigator (LK) in the second stage [46]. NJH and LK met and agreed on a deductive coding framework before its systematic application to all transcripts. The framework was inductively adapted as new codes emerged (indexing). Codes were summarised in a matrix in an Excel worksheet. Next, each theme and codes were allocated a column (charting). Data were colour-coded in an iterative process to identify emerging themes. Regular researcher triangulation meetings were held by NJH and LK to review and shape the analyses [46].

### 3.7. Ethical considerations

This study was approved by the Science, Technology, Engineering and Mathematics Ethical Review Committee, University of Birmingham (ERN_23–1041) and Barnardo’s Research Ethics Committee. All participants were provided with participant information sheets and signed a written informed consent form before any data collection activity commenced. In the case of any emotional unrest during the interviews and focus group, the local charity has a referral process to support participants. A designated staff member from the charity was available as trusted support before, during, and after each session for participants.

## 4. Findings

### 4.1. Participants characteristics

The final sample consisted of 15 participants, including 12 women who engaged in semi-structured interviews and three women who participated in a focus group discussion. All three focus group participants spoke English fluently. Of the interviewees, nine spoke English fluently, while three who do not speak English as their first language were interviewed with the assistance of a Punjabi interpreter. All participants were recent postpartum women at the time of the study and were accessing the services of the local charity. The majority of participants identified themselves as Indian, while the remaining participants reported ethnic identities including, Black African, Bangladeshi, Pakistani, Afghan, and Black Caribbean.

### 4.2. Overview

Following inductive coding, four organising themes were created, as described in what follows. Illustrative quotes relevant to each theme are presented.

**Theme 1: Mixed Experiences and Perceptions of Perinatal Mental Health Screening Tools**

The first organising theme was mixed experiences and perceptions of perinatal mental health screening tools, which is interconnected with other organising themes. Within this theme, participants responses are categorised into two groups: Screening tools experienced as emotional questionnaires and emotional readiness to engage with screening tools.

**Theme 1.1: Screening Tools Experienced as Emotional Questionnaires**

A significant theme that emerged from the interviews and focus group discussions was the concept of emotions. Participants described their experiences of mental health screening tools as answering questions about their feelings.

*“…. it* [screening tools] *was about answering are you happy? How are you feeling and that’s it*” (Interview participant 1).

“*During all of my appointments and other stuff with the midwife, they would ask me one question or two questions. So, the one I remember mainly is, are you OK at home? …. and the other one is how are you feeling*?” (Focus Group participant 1).

Furthermore, most participants found the assessment tools to be straightforward, effective, and viewed them as tools for discussing about their emotional state.

“*The questions are quite straightforward because it’s just like how you felt recently, and you felt like harming yourself*” (Interview participant 6).

“*I think it* [screening tools] *is effective, but they can do more as in talking though…*” (Interview participant 5).

**Theme 1.2: Emotional Readiness to Engage with Screening Tools**

This theme highlights how women’s emotional states during mental health screening were linked to their experiences and perceptions of the assessment tools and willingness to answer question on the scale. Women who reported being in a “happy place” or who had support from family members and partners were more comfortable disclosing their feelings during screening interactions or answering question from the assessment tools. For these participants, the existence of reliable social support appeared to enhance their confidence in responding openly to screening questions, as their immediate emotional needs were already being met within their personal networks.

“*When she* [midwife] *asked the question, I was in a happy place…. I had the support network in place. So, I was happy to answer that question*” (Interview participant 1).

“*Like I said, I had a supportive family. They were always there for me…. My partner was also supportive as well so…. yeah, I will be 100% able to answer it*” (Interview participant 4).

However, for others, readiness to disclose or answer questions from the tools were influenced by the degree of trust placed in healthcare professionals. Some participants reported feelings of vulnerability and discomfort with answering personal questions and feelings of worry and fear about disclosure. They described uncertainty regarding how personal information might be used or shared, expressing concerns about confidentiality, potential disclosure to family members, and the broader implications of revealing mental health difficulties as stated by one participant:

“*Like your trust in talking to that person.... how they’re going to share it or who they’re going to share it with. I think it* [feeling of worry]*‘d be with how the information is shared, like what would happen with it once if you did say something? Would they pass it on to like family or would it go through to like a doctor?*” (Interview participant 11).

**Theme 2: Factors Shaping Women’s Perceptions and Engagement with Perinatal Mental Health Screening Tools: Barriers and facilitators**

The second overarching theme was factors shaping women’s perceptions and engagement with perinatal mental health screening tools. This theme indicates that participants in both focus groups and interviews expressed a variety of factors, which were categorised into four interconnected concepts: Provider communication and lack of clarity; time constraints and provider interest; language barriers; and fear of disclosure outcomes undermine responses to screening questions.

**Theme 2.1: Provider communication and lack of clarity**

Answering questions from the tools were described as having a casual chat or routine conversation with providers and this was indicted as a barrier to revealing mental health concerns during screening. One participant described the tools as scripted questionnaire:

“*It was just like a chat…. It didn’t feel like something that was just like a tick box. He* [The general practitioner] *screened me by looking at my mannerisms, like how, how I sat down and how I was talking…. And then he wanted to watch how I would talk to him, how I would see the baby, and he did go through, like, a scripted question on the computer*” (Focus Group participant 2).

Other participants indicated that they did not perceive a necessity to provide feedback regarding the questions posed on the tools, nor did they believe there was a need to reveal any perinatal mental health concerns. They believed that a lack of clarity and information regarding the purpose, process, and use of screening tools limited their engagement with mental health screening scales:

“*So, I feel it’s like a normal chat and that feedback is not needed as such, it’s felt like a general chat. They didn’t say this is the reason why we ask. No reason for asking those questions*” (Interview participant 1).

“*No, she asked me how I was feeling but she didn’t say oh, so this is what we’re going to do, and this is how we gonna do it*” (Interview participant 2).

“*If you go in there and this person just brings all this form* [screening tools] *at you and then didn’t even tell you what your results were or whatever, you’re gonna think “well, what was all that about, what did you just do*” (Interview participant 6).

Additionally, the women expressed that they were unfamiliar with mental health screening tools and their scores and had not received any information about these tools prior to screening, for example:

“*To me, this is the first time am hearing about maternal mental health screening tool. This is the first time*” (Interview participant 4).

“*I was never told what the score means to me. Do you know what I mean? No, never explain the purpose to say..... we’ll just go through the questions, and at the end, he* [Doctor] *didn’t say the score means this*” (Interview participant 6).

Provider communication and clarity were closely associated with participant’s perception of screening tools. Participants reported that screening tools were acceptable and potentially effective when their purpose and content were clearly explained. Clear communication about the screening process and questions were viewed as essential for meaningful engagement. As one participant stated:

“*So basically, it* [screening tool] *is fine with me as long as it’s explained what is ask*” (Interview participant 7).

**Theme 2.2: Time constraints and provider interest**

Competing priorities from providers and time constraints hinders effective engagement with screening tools. Women emphasised the importance of allocating sufficient time for maternal mental health assessment to ensure the effectiveness of screening tools, as the process felt rushed:

“*Oh! Yeah, this appointment is ending now......there’s a whole waiting room full of people…..yeah, it felt probably a bit rushed*” (Interview participant 2).

*“.... if the people like midwives and the doctors and everybody, if they can give more time to the ladies who are pregnant and stuff to like, make the appointment and see them and then discuss with them... like few hours to spend with them to ask the problems and to ask how you feel in the home*” (Interview participant 9).

A focus group participant emphasised the importance of allowing adequate time for formal screening while sharing her experience of being examined for postnatal depression.

*“…… And I felt a bit of a relief that he took time out and did it bit more professionally than I could logically think in my brain*” (Focus group participant 2).

Lack of interest was identified as a barrier shaping perceived effectiveness of screening tools. Some argued that the implementation of perinatal mental health screening tools should prioritise women’s mental health rather than focusing primarily on their babies and the progress of their pregnancies. As one participant noted:

“*I felt like he* [Doctor] *was asking the right questions, but not in the right sense, like here my questions was based on the newborn…It was all about the baby, baby, baby*” (Interview participant 6).

Another interviewee mentioned not disclosing her postnatal mental health concerns or honestly answering questions from the tools due to a lack of interest and attention from a provider. This is supported by the quote below:

“*I didn’t feel like telling them* [health professionals] *because they’ll just ask a question and then they’ll just leave it and carry on with like looking at the baby and stuff*” (Interview participant 5).

**Theme 2.3: Language barriers**

The perceived barriers shaping perceptions of the effectiveness of perinatal mental health screening tools for women from minority ethnic groups also arise from issues of language difficulties. Participants mentioned specific language barriers and challenging wordings. They highlighted difficulty understanding words with complex and multiple meanings. Exemplar quote includes:

“*I think they* [women from the Asian community] *need to be aware of it* [screening tools] *in their language. You know whether it is, you know, whether it’s Punjabi, Urdu or Polish, it doesn’t matter*” (Interview participant 3).

Many women who spoke about needing explanations for mental health assessment tools also expressed that the use of elaborate words is unnecessary. They noted this as a challenge to understanding the questions on the tools and two women highlighted this concern by discussing the challenges words such as “*edge*” and “*anxious”*, both from the Generalized Anxiety Disorder Questionnaire (GAD-7), presents:

*“...depends if they* [Bangladeshi women] *have an interpreter, do they know what on edge means? Because on edge, it’s like a phrase, isn’t it? So, does that translate back to what you mean it to mean? I don’t think there’s a word for anxiety because am Bangladeshi”* (Interview participant 2).

“*Anxiety I can relate to, I know what that means, but words like “edgy” wouldn’t mean anything….. anxiety, depression I know, I don’t know* [edgy]*. Anxious, not sure about that, nervous I know*” (Interview participant 7).

Being on edge is a ‘polysemous’ phrase with multiple meanings. Moreover, participants felt that the use of polysemous words affected their comprehension and responses to the Whooley questions and Edinburgh Postnatal Depression Scale (EPDS).

*“I think that they’re quite easy. So, I think some questions are fine because they are very straightforward, but those ones that have multiple words and multiple meanings they can be more difficult”* (Interview participant 2).

*“Use words that aren’t, don’t have so many meanings. Just the basic word that is self- explanatory and we’re aware of or whatever your language you will understand”* (Interview participant 6).

**Theme 2.4 Fear of disclosure outcomes undermine responses to screening questions**

Another element was fear of the potential consequences of disclosing mental health concerns, which is associated with limited understanding of screening tools and their intended purpose. One interviewee referenced a question from the EPDS that exemplified this concern:

*“.... there are questions when you ask and then it leads to another thing. Why will you blame yourself? Is it going to affect your children? …. So, there are questions that are better not to be answered, not to even think about it”* (Interview participant 8).

Participants’ perceptions of the assessment tools were shaped by their experiences during screening process. For example, one focus group participant expressed uncertainty about how her responses would be used and voiced concerns about potential negative consequences, such as judgment or involvement of social services. These apprehensions contributed to viewing the tools as ineffective, illustrating how fear of disclosure outcomes can undermine trust and engagement with perinatal mental health screening tools.

*“…… but I don’t think it’s* [screening tool] *effective because even when I had my break down, they said to me has there been any social services involvement and straight away I was like, oh, great, that’s it and I’ll just kind of put up a wall*” (Focus group participant 3).

Participants’ perceptions of perinatal mental health screening tools were also influenced by concerns about privacy. Several women described feeling unable to respond honestly when others were present during the screening process. One participant explained:

*“…and my partner was there, and this health visitor asked… I couldn’t speak up because I’m scared that he is there, and I can’t tell the truth*” (Interview participant 4).

**Theme 3: Lack of Continuity, Follow-up, and Clear Pathways Hinders Women’s Engagement with Mental Health Screening Tools**

Across interviews, participants consistently described significant gaps in follow-up, continuity of care, and absence of clear pathway, which left many feeling unsupported during perinatal screening. Women expressed a strong desire for proactive check-ins, such as a phone call or brief follow-up after appointments where distress was noted.

“*I think if I did get a phone call or something to say…. Yeah. Any follow-up to kind of say ohh, you know, you were feeling upset at this appointment, how are you feeling now? Yeah, I think that would have been appreciated at the time*” (Interview participant 2)

“*I would have appreciated a call back at least to say…. if not an appointment just if am still feeling the same, do you need support or are you in a good place?*” (Interview participant 7).

Several women articulated a lack of knowledge regarding available services or appropriate points of contact, which prevented them from acting on intentions to seek help or engaging with screening tools. This absence of clear pathways meant that perinatal mental health concerns went unaddressed, even when women recognised a need for support. One woman described the challenges faced by women in her community, while another verbalised her struggles in disclosing her perinatal mental health concerns.

“*Because a lot of people and a lot of women don’t know, don’t have a clue, they don’t know that they can get help from so and so person*” (Interview participant 5).

“*I contemplated the idea of speaking to somebody about my mental health, but because I didn’t know what to do, I didn’t know how to go about it, I never did*” (Interview participant 7).

Participants reported substantial delays in initial contact with maternity services, often waiting several weeks for a first appointment or scan. These delays were described as occurring during periods of heightened vulnerability and were associated with increased anxiety and a perceived lack of support in early pregnancy, which participants felt could undermine engagement with perinatal mental health screening tools. As these participants described:

“*I couldn’t even get hold of anyone to talk to in the first place and my scan came much more later…..I was surprised that there’s kind of until your first scan, there’s no contact with anyone ….I feel like that’s the moment when you’re most vulnerable*” (Interview participant 2).

“... *I think I was four weeks* [pregnant] *and I contact them* [health care provider]*, they have to wait till eight weeks before I get appointment for the first scan. I was so stressed*” (Interview participant 4).

In addition, participants emphasised that emotional and hormonal fluctuations are present throughout the pregnancy trajectory, including in later stages, yet routine enquiry or screening for mental health was often limited to specific time points. One woman felt that such episodic approaches undermine engagement with perinatal mental health screening tools.

*“…. whatever month you are a woman still goes through like a hormonal change. I feel like even then they should ask* [screen]*. I’m talking about personal experience towards the end of my pregnancy; my hormones were all over the place*” (Interview participant 5).

**Theme 4: Recommendations for Improvement**

As previously mentioned, women’s experiences and perceptions highlighted that engagement with perinatal mental health screening tools is strongly influenced by the emotional, cultural, and structural aspects of screening delivery. Their accounts point to the importance of cultural and language accessibility, continuity in care, and flexibility in how questions are framed to enable honest disclosure. This theme contained three discrete but interconnected elements: Cultural and language accessibility enables engagement with screening tools; Consistency of care as a foundation for trust and disclosure; and Flexibility in screening tools format supports honest response.

### 4.3. Cultural and language accessibility enables engagement with screening tools

All interviewees, irrespective of their ethnic background or English proficiency, acknowledged the critical role of an interpreter when applying screening tools for women whose first language is not English. The provision of interpretation services was identified as crucial for equitable and meaningful engagement with screening tools. Participants appreciated when support was tailored to their cultural and language needs, noting that having access to an interpreter facilitated comprehension and reduced communication barriers. One interviewee stated:

“*I went to every appointment with her and then when the health visitor came, she always had a Punjabi interpreter……. The fact that they was explaining to her what they were asking through the interpreter, that made it easy …..*” (Interview participant 10).

Participants highlighted the importance of responding to screening questions in their local language, with the assistance of an interpreter, as it enhanced their understanding and allowed for more honest answers.

“*Possibly it could be having somebody like a translator or someone in the room that could speak the language of the lady who’s there, rather than just in English, and then the lady might just assume she can only say yes. If you had someone there that could speak her language, she might feel a bit more comfortable with opening up*” (Interview participant 11).

### 4.4. Consistency of care as a foundation for trust and disclosure

Participants consistently emphasised the value of seeing the same health professional throughout the perinatal period. Consistency fostered familiarity and trust, and confidence to answer question from screening tools. Women described greater willingness to disclose mental health concerns when screening tool was implemented by a practitioner with whom they had an established relationship.

“*I think it’s ideal to see the same person* [health professional] *because they know your history, or they know..*.” (Interview participant 3).

“*Having the same midwife made me feel comfortable. Can’t share my personal things to another person*” (Interview participant 12).

Conversely, when screening was undertaken by a practitioner they had not previously met, participants described feeling less comfortable and more guarded in their responses. Lack of consistency appeared to undermine psychological safety and inhibit honest answers to questions on mental health screening tools.

“*The midwife I saw was like sort of straight after I had given birth to him* [baby]. *She* [midwife] *asked me those questions, but she was a stranger to me, and I guess, yeah, I didn’t feel comfortable to…*” (Interview participant 2).

### 4.5. Flexibility in screening tools format supports honest response

Participants revealed mixed experiences with the structure and language of screening tools. While some found straightforward, simple questions easy to engage with, others felt constrained by closed-ended or multiple-choice formats. Participants expressed concern that standardised options did not always reflect their actual feelings, potentially leading to inaccurate assessments:

“*Rather than having multiple questions to tick, it’s better as an open question and …. you don’t have to pick something within that box*” (Interview participant 7).

Many preferred open-ended questions that enabled them to describe their experiences in their own words, suggesting that more flexible screening formats may support deeper and more honest disclosure.

“*....... a box for you to write in there how you been feeling because you might not have been feeling nervous, anxious, or edgy, and then you’re gonna put NO or Not at all and then they’re gonna think this person is ok. So, you make it more of an open question. Don’t give people options to choose from. You might not have the opinion any of those three*” (Interview participant 6).

## 5. Discussion

We conducted a qualitative study to explore how fifteen women from minority ethnic groups in the UK perceive and experience screening tools for identifying mental health issues during the perinatal periods. Findings showed that there were four key themes shaping the experiences of mental health screening tools among this under-served group: Mixed experiences and perceptions of perinatal mental health screening tools; Factors shaping women’s perceptions and engagement with perinatal mental health screening tools: barriers and facilitators; Lack of continuity, early support, and clear pathways hinders women’s engagement with mental health screening tools; and Recommendations for improvement. These themes can inform service delivery by assisting in the design and implementation of screening tools, aiding health practitioners in effectively utilising these screening tools for women from ethnic minority communities. The findings suggest that the majority of women in this study perceived the EPDS, GAD-7, and the Whooley questions primarily as tools for exploring feelings and value the opportunity to express their emotions. Previous studies have emphasised that screening can serve as a gateway for dialogue, enabling women to articulate concerns that might otherwise remain unspoken [47,48].

This sense of support is particularly significant for ethnic minority populations, including migrants, who often face barriers such as language differences, cultural stigma, and limited access to mental health services [25]. Specifically, women from ethnic minority backgrounds, including those who are migrants, frequently characterised perinatal mental health screening as a valuable mechanism for articulating their emotional experiences to healthcare professionals [49–51]. By creating a space for emotional disclosure, screening tools may help mitigate these barriers and foster trust between patients and providers. However, this interpretation raises questions whether women feel emotionally prepared to engage with mental health screening tools and provide truthful answers. Our findings reinforce existing evidence that women’s perceptions of perinatal mental health screening tools are closely influenced by their emotional state during the screening process. This aligns with prior research suggesting that emotional readiness can shape both the acceptability and perceived effectiveness of screening [32,48]. Importantly, although women from this study generally reported feeling comfortable engaging with screening questions and discussing mental health concerns with practitioners, their emotional state at the time of screening emerged as a critical factor influencing engagement and response patterns. This underscores the need for healthcare providers to adopt approaches that are sensitive to emotional variability during screening, as these dynamics may ultimately affect the quality of care and support women receive. In the second qualitative theme, findings suggest that informal communication from providers and lack of clarity, associated with time constraints, poor provider interest, and language barriers influence women’s engagement with perinatal mental health screening tools and consequently, impacts their perceived effectiveness of these tools. Participants in this study said they did not realise they were being screened for mental health concerns and described their experiences as having a causal chat. This perception aligns with previous research indicating that although the informality of screening process could potentially support with disclosure of mental health challenges through enhancing rapport, it is also a weakness because women did not have enough information to understand they were experiencing screening [52]. Similarly, studies indicate that inattentive and dismissive attitudes among healthcare providers may exacerbate patients’ reluctance to disclose information honestly [20,52]. Specifically, providers frequently failed to explain the purpose and intended use of the screening tools and did not communicate the results to patients [52]. This absence of structured communication left women uncertain about the relevance and outcomes of the screening, reinforcing feelings that the process was superficial rather than meaningful. Such findings highlight the critical role of provider communication in shaping perceptions of screening tools and suggest that inadequate explanation and feedback can undermine trust and reduce the perceived value of these perinatal mental health assessment tools. To address this, women may benefit from more explanation about screening tools. Transitioning the implementation of screening tools from an informal to a formal setting may help women understand the importance of disclosing mental health concerns to their healthcare professionals, although the formality of the process could also inhibit disclosure, too, due to concerns identified in the literature review. This finding also underscores the critical need for healthcare providers to receive training in structured communication protocols to build confidence in ensuring effective and consistent mental health care delivery [53]. It also emphasised the importance of dedicating enough time and attention to maternal mental health to effectively utilise its screening tools and encourage honest disclosure of perinatal mental health problems.

A significant finding is that women raised concerns around issues of language difficulties and challenging wordings on screening tools. They highlighted issues in comprehending certain terms and stressed difficulty understanding polysemous words and phrases with complex and multiple meanings. To exemplify, the women in this study highlighted the difficulty of translating specific words such as “edge” into other languages, which often leads to losing the meaning and essence of the original text. Santiago and Figueiredo [29] and Nilaweera et al [30] also emphasise the impact of language barriers on access and engagement with services. This reflects the importance of effective communication, language proficiency and sensitivity when designing measures for mental health care [54]. According to Verschuuren et al [20] and Watson et al [25], language barriers, insufficient translated resources, and concerns regarding interpreter confidentiality significantly impede comprehension and honest disclosure during perinatal mental health screening. There are several potential ways to address comprehension. One is that the wording of the scales could be changed to be more interpretable to those from under-served groups. Another is that interpreters who are trained in mental health could help facilitate effective screening [20,32]. Participants in this study highlighted the critical role of an interpreter during perinatal mental health screening. Professional interpreters can act as mental health screening champions to facilitate effective communication and build trust between women and health professionals and improve clinical care [55], though concerns about confidentiality and accuracy remain. Beyond wording, the content of the items in some of the scales used in this study were concerning to women. The sensitive EPDS items regarding self-harm were noted as concerning, particularly without adequate information about the potential consequences of disclosing such personal issues. Participants described uncertainty about how their responses would be used and expressed apprehension about possible negative outcomes, such as judgment or involvement of social services. This uncertainty often shape women’s perceived ineffectiveness of the tools, leading to apprehension about revealing mental health concerns. Women reported that insufficient explanation from providers left them unsure about the role of screening tools, which heightened anxiety and influenced their willingness to answer questions honestly. These findings echo previous research suggesting that inadequate communication during screening can undermine trust and reduce engagement [52]. This highlights the need for healthcare professionals to ensure confidentiality and build trust when implementing mental health screening tools with this group of women. These findings emphasise the need for comprehensive provider training in culturally competent communication and mental health care [20]. Future research should prioritise the development of clear guidance, culturally sensitive communication strategies, and simplified versions of screening tools to improve women’s perceptions of perinatal mental health assessments, with particular attention to the needs of ethnic minority populations.

In third qualitative theme, participants emphasised the importance of continuity in care and the need for flexibility in framing questions to facilitate honest disclosure. Previous research recommends involving professionals from ethnic minority backgrounds and adopting continuity-of- care models as strategies to improve understanding and enhance women’s engagement with perinatal mental health screening tools [20,25]. These approaches aim to address communication barriers and foster trust, which are critical for improving screening effectiveness among diverse populations. The present findings also indicate a need to incorporate open-ended questions within perinatal mental health screening tools to provide women with greater flexibility in expressing their emotions. Closed-ended formats, while efficient, may limit nuanced responses and fail to capture the complexity of patients’ experiences, particularly among those who struggle to interpret standardised items [56]. Indeed, respondents in general mental health surveys have been shown to provide richer, more nuanced data when open-ended items are included, even though this may come at the expense of missing responses [57]. In perinatal care, open-ended questions can foster patient-centred communication and enhance disclosure by allowing respondents to articulate concerns in their own language, a critical factor for populations facing linguistic or cultural barriers, such as ethnic minority and underserved groups [47]. Integrating open-ended prompts alongside structured screening tools may be especially beneficial for ethnic minority women and those from migrant backgrounds, who often face linguistic and conceptual barriers when engaging with rigid screening formats [20,25]. Healthcare providers should consider adopting hybrid screening models that combine standardised tools with open-ended prompts. This strategy can promote trust, acceptability of perinatal mental health assessment tools, encourage emotional disclosure, and ensure that screening tools captures culturally nuanced experiences. Further studies are needed to evaluate the effectiveness of incorporating open-ended questions into existing screening tools and to explore how these modifications influence engagement and diagnostic accuracy.

## 6. Strengths and limitations

A significant strength of this study was the recruitment of women from under-served communities, engaging them in in-depth and sensitive discussions, and providing a platform for the voices of these unique ethnic groups. Their contributions facilitated the identification of four key themes to consider when administering maternal mental health screening tools among women from ethnically diverse communities. However, this study is not without limitations. As the majority of participants self- identified as being from Asian backgrounds, the perspectives captured in this study may not be generalisable to women from other minority ethnic communities within the region. Future research is therefore needed to explore how these findings resonate with other under-served groups in new cultures and contexts. It is also important to consider screening in the context of how other wider social and cultural barriers affect perinatal mental health concerns as perceived by women from minority ethnic communities.

## 7. Conclusions and recommendations

This qualitative study has shown that women from minority ethnic communities in the UK have a diverse variety of experiences with perinatal mental health screening tools. Their experiences can be used to inform the optimal design and delivery of these tools for women from ethnic minority communities. It is important to formally inform women about mental health and its screening tool and process, specifically among women from underserved communities and ethnically diverse backgrounds to help address cultural barriers. Mental health professionals should explain the purpose and benefits of screening in local languages using familiar, clear words, and interpreters if required. Perinatal mental health screening tools should be culturally adapted to address cultural barriers. There is a need to consider integrating open-ended prompts alongside structured screening tools, to enhance both the acceptability and accuracy of perinatal mental health assessments, while also promoting trust and engagement. These initiatives may significantly enhance perceptions of screening tools among women from minority ethnic groups. The findings of this study align with the Maternal Mental Health Alliance’s commitment to ensuring equitable access to quality perinatal mental health care by underscoring the necessity of culturally adapted screening protocols, culturally sensitive and clear communication strategies, and emotionally supportive environments in delivering effective perinatal mental health support for women from ethnic minority ethnic backgrounds [58].

## Supporting information

S1 Appendix A brief description of the Edinburgh Postnatal Depression Scale (EPDS) [15], Generalized Anxiety Disorder Assessment (GAD-7) [16] and Whooley questions [18]. (PDF)

S2 Appendix Interview and focus group topic guide. (PDF)

## References

[1] O’Hara MW, Wisner KL. Perinatal mental illness: definition, description and aetiology. Best Pract Res Clin Obstet Gynaecol. 2014;28(1):3–12. doi: 10.1016/j.bpobgyn.2013.09.002 24140480 PMC7077785

[2] Howard LM, Khalifeh H. Perinatal mental health: a review of progress and challenges. World Psychiatry. 2020;19(3):313–27. 32931106 10.1002/wps.20769 PMC7491613

[3] Caffieri A, Gómez‐Gómez I, Barquero‐Jimenez C, De‐Juan‐Iglesias P, Margherita G, Motrico E. Global prevalence of perinatal depression and anxiety during the COVID‐19 pandemic: An umbrella review and meta‐analytic synthesis. Acta obstetricia et gynecologica Scandinavica. 2024;103(2):210–24. 10.1111/aogs.14740 PMC10823409 38113292

[4] Gelaye B, Rondon MB, Araya R, Williams MA. Epidemiology of maternal depression, risk factors, and child outcomes in low-income and middle-income countries. Lancet Psychiatry. 2016;3(10):973–82. doi: 10.1016/S2215-0366(16)30284-X 27650773 PMC5155709

[5] National Institute for Clinical Excellence. Antenatal and postnatal mental health: clinical management and service guidance. Clinical Guideline No.192. 2020 [cited 2023 May 31]. Available from: https://www.nice.org.uk/guidance/cg192

[6] Staneva A, Bogossian F, Pritchard M, Wittkowski A. The effects of maternal depression, anxiety, and perceived stress during pregnancy on preterm birth: A systematic review. Women Birth. 2015;28(3):179–93. doi: 10.1016/j.wombi.2015.02.003 25765470

[7] Waters CS, Hay DF, Simmonds JR, van Goozen SHM. Antenatal depression and children’s developmental outcomes: potential mechanisms and treatment options. Eur Child Adolesc Psychiatry. 2014;23(10):957–71. doi: 10.1007/s00787-014-0582-3 25037152

[8] Price DAM, Middleton MM, Matthey AAPS, Goldfeld PS, Kemp PL, Orsini MF. A comparison of two measures to screen for mental health symptoms in pregnancy and early postpartum: the Matthey Generic Mood Questionnaire and the Depression, Anxiety, Stress Scales short-form. J Affect Disord. 2021;281:824–33. doi: 10.1016/j.jad.2020.11.055 33334609

[9] Felker A, Patel R, Kotnis R, Kenyon S, Knight M, MBRRACE-UK Collaboration. Saving Lives, Improving Mothers’ Care Compiled Report - Lessons learned to inform maternity care from the UK and Ireland Confidential Enquiries into Maternal Deaths and Morbidity 2020-22. University of Oxford; [cited 2024 Nov 20]. Available from: https://www.npeu.ox.ac.uk/mbrrace-uk/reports/maternal-reports/maternal-report-2020-2022

[10] O’Connor E, Rossom RC, Henninger M, Groom HC, Burda BU. Primary Care Screening for and Treatment of Depression in Pregnant and Postpartum Women: Evidence Report and Systematic Review for the US Preventive Services Task Force. JAMA. 2016;315(4):388–406. doi: 10.1001/jama.2015.18948 26813212

[11] Al-Abri K, Edge D, Armitage CJ. Prevalence and correlates of perinatal depression. Soc Psychiatry Psychiatr Epidemiol. 2023;58(11):1581–90. doi: 10.1007/s00127-022-02386-9 36646936 PMC9842219

[12] Prady SL, Endacott C, Dickerson J, Bywater TJ, Blower SL. Inequalities in the identification and management of common mental disorders in the perinatal period: An equity focused re-analysis of a systematic review. PLoS One. 2021;16(3):e0248631. doi: 10.1371/journal.pone.0248631 33720994 PMC7959342

[13] World Health Organization. WHO recommendations on maternal and newborn care for a positive postnatal experience. World Health Organization; 2022 Mar 29 [cited 2024 Dec 21]. Available from: https://www.who.int/publications/i/item/9789240045989 35467813

[14] Sambrook Smith M, Cairns L, Pullen LSW, Opondo C, Fellmeth G, Alderdice F. Validated tools to identify common mental disorders in the perinatal period: A systematic review of systematic reviews. J Affect Disord. 2022;298(Pt A):634–43. doi: 10.1016/j.jad.2021.11.011 34763033

[15] Cox JL, Holden JM, Sagovsky R. Detection of postnatal depression. Development of the 10-item Edinburgh Postnatal Depression Scale. Br J Psychiatry. 1987;150:782–6. doi: 10.1192/bjp.150.6.782 3651732

[16] Swinson RP. The GAD-7 scale was accurate for diagnosing generalised anxiety disorder. Evid Based Med. 2006;11(6):184. doi: 10.1136/ebm.11.6.184 17213178

[17] Spitzer RL, Kroenke K, Williams JB, Löwe B. A brief measure for assessing generalized anxiety disorder: the GAD-7. Arch Int Med. 2006;166(10):1092–7. 16717171 10.1001/archinte.166.10.1092

[18] Whooley MA, Avins AL, Miranda J, Browner WS. Case-finding instruments for depression. Two questions are as good as many. J Gen Intern Med. 1997;12(7):439–45. doi: 10.1046/j.1525-1497.1997.00076.x 9229283 PMC1497134

[19] Donnelly O, Leavey G. Screening Tools for Mental Disorders Among Female Refugees: a Systematic Review. J Child Adolesc Trauma. 2021;15(2):209–19. doi: 10.1007/s40653-021-00375-9 35600514 PMC9120328

[20] Verschuuren AEH, Soldati E, Stekelenburg J, Jong EIF, Postma IR. Screening instruments for antenatal and postpartum mental health disorders in migrant women: a systematic review. Arch Womens Ment Health. 2025;28(2):219–44. doi: 10.1007/s00737-024-01552-z 40042676 PMC11991996

[21] Office for National Statistics. Population of England and Wales [Internet]. London: Office for National Statistics; 2022 Dec 22 [updated 2024 May 21; cited 2026 Jan 3]. Available from: https://www.ethnicity-facts-figures.service.gov.uk/uk-population-by-ethnicity/national-and-regional-populations/population-of-england-and-wales/latest/

[22] Watson E, Evans SJ. An example of cross-cultural measurement of psychological symptoms in post-partum mothers. Soc Sci Med. 1986;23(9):869–74. doi: 10.1016/0277-9536(86)90215-7 3798166

[23] Yoshida K, Marks MN, Kibe N, Kumar R, Nakano H, Tashiro N. Postnatal depression in Japanese women who have given birth in England. J Affect Disord. 1997;43(1):69–77. doi: 10.1016/s0165-0327(96)01419-x 9127832

[24] Fuggle P, Glover L, Khan F, Haydon K. Screening for postnatal depression in Bengali women: Preliminary observations from using a translated version of the Edinburgh Postnatal Depression Scale (EPDS). J Reprod Infant Psychol. 2002;20(2):71–82. doi: 10.1080/02646830220134603

[25] Watson H, Harrop D, Walton E, Young A, Soltani H. A systematic review of ethnic minority women’s experiences of perinatal mental health conditions and services in Europe. PLoS One. 2019;14(1):e0210587. doi: 10.1371/journal.pone.0210587 30695019 PMC6351025

[26] Waqas A, Koukab A, Meraj H, Dua T, Chowdhary N, Fatima B, et al. Screening programs for common maternal mental health disorders among perinatal women: report of the systematic review of evidence. BMC Psychiatry. 2022;22(1):54. doi: 10.1186/s12888-022-03694-9 35073867 PMC8787899

[27] Webb R, Uddin N, Ford E, Easter A, Shakespeare J, Roberts N, et al. Barriers and facilitators to implementing perinatal mental health care in health and social care settings: a systematic review. Lancet Psychiatry. 2021;8(6):521–34. doi: 10.1016/S2215-0366(20)30467-3 33838118

[28] Smith MS, Lawrence V, Sadler E, Easter A. Barriers to accessing mental health services for women with perinatal mental illness: systematic review and meta-synthesis of qualitative studies in the UK. BMJ Open. 2019;9(1):e024803. doi: 10.1136/bmjopen-2018-024803 PMC6347898 30679296

[29] Santiago M da CF, Figueiredo MH. Immigrant women’s perspective on prenatal and postpartum care: systematic review. J Immigr Minor Health. 2015;17(1):276–84. doi: 10.1007/s10903-013-9915-4 24052479

[30] Nilaweera I, Doran F, Fisher J. Prevalence, nature and determinants of postpartum mental health problems among women who have migrated from South Asian to high-income countries: a systematic review of the evidence. J Affect Disord. 2014;166:213–26. doi: 10.1016/j.jad.2014.05.021 25012434

[31] Jankovic J, Parsons J, Jovanović N, Berrisford G, Copello A, Fazil Q, et al. Differences in access and utilisation of mental health services in the perinatal period for women from ethnic minorities-a population-based study. BMC Med. 2020;18(1):245. doi: 10.1186/s12916-020-01711-w 32912196 PMC7488566

[32] Willey SM, Blackmore RP, Gibson-Helm ME, Ali R, Boyd LM, McBride J, et al. “If you don’t ask … you don’t tell”: Refugee women’s perspectives on perinatal mental health screening. Women Birth. 2020;33(5):e429–37. doi: 10.1016/j.wombi.2019.10.003 31759865

[33] Bayrampour H, McNeil DA, Benzies K, Salmon C, Gelb K, Tough S. A qualitative inquiry on pregnant women’s preferences for mental health screening. BMC Pregnancy Childbirth. 2017;17(1):339. doi: 10.1186/s12884-017-1512-4 28974195 PMC5627476

[34] O’Brien BC, Harris IB, Beckman TJ, Reed DA, Cook DA. Standards for reporting qualitative research: a synthesis of recommendations. Acad Med. 2014;89(9):1245–51. doi: 10.1097/ACM.0000000000000388 24979285

[35] Gill P, Stewart K, Treasure E, Chadwick B. Methods of data collection in qualitative research: interviews and focus groups. Br Dent J. 2008;204(6):291–5. doi: 10.1038/bdj.2008.192 18356873

[36] Sandwell Metropolitan Borough Council. Sandwell Authority Monitoring Report 2023-2024 [Internet]. 2024 [cited 2024 Dec 20]. Available from: https://www.sandwell.gov.uk/downloads/download/478/sandwell-authority-monitoring-report

[37] Sandwell Metropolitan Borough Council. Town Profiles [Internet]. 2024 [cited 2024 Jan 12]. Available from: https://www.sandwelltrends.info/town-profiles/

[38] Palinkas LA, Horwitz SM, Green CA, Wisdom JP, Duan N, Hoagwood K. Purposeful sampling for qualitative data collection and analysis in mixed method implementation research. Adm Policy Ment Health Ment Health Serv Res. 2015;42:533–44. 10.1007/s10488-013-0528-y PMC4012002 24193818

[39] Conneely M, Packer KC, Bicknell S, Janković J, Sihre HK, McCabe R, et al. Exploring Black and South Asian women’s experiences of help-seeking and engagement in perinatal mental health services in the UK. Front Psychiatry. 2023;14:1119998. doi: 10.3389/fpsyt.2023.1119998 37077277 PMC10109459

[40] Guest G, Namey E, Chen M. A simple method to assess and report thematic saturation in qualitative research. PLoS One. 2020;15(5):e0232076. doi: 10.1371/journal.pone.0232076 32369511 PMC7200005

[41] Yuill C, Sinesi A, Meades R, Williams LR, Delicate A, Cheyne H, et al. Women’s experiences and views of routine assessment for anxiety in pregnancy and after birth: A qualitative study. Br J Health Psychol. 2024;29(4):958–71. doi: 10.1111/bjhp.12740 38955505

[42] Holland R. Reflexivity. Hum Relat. 1999;52(4):463–84.

[43] Olmos-Vega FM, Stalmeijer RE, Varpio L, Kahlke R. A practical guide to reflexivity in qualitative research: AMEE Guide No. 149. Med Teach. 2023;45(3):241–51. 10.1080/0142159X.2022.2057287 35389310

[44] Furber C. Framework analysis: a method for analysing qualitative data. Afr J Midwifery Women’s Health. 2010;4(2):97–100. doi: 10.12968/ajmw.2010.4.2.47612

[45] Collaço N, Wagland R, Alexis O, Gavin A, Glaser A, Watson EK. Using the Framework Method for the Analysis of Qualitative Dyadic Data in Health Research. Qual Health Res. 2021;31(8):1555–64. doi: 10.1177/10497323211011599 33980102 PMC8278550

[46] Donkoh S. Application of triangulation in qualitative research. JABB. 2023;10(1):6–9. doi: 10.15406/jabb.2023.10.00319

[47] Dennis C-L, Chung-Lee L. Postpartum depression help-seeking barriers and maternal treatment preferences: a qualitative systematic review. Birth. 2006;33(4):323–31. doi: 10.1111/j.1523-536X.2006.00130.x 17150072

[48] Kingston D, Austin M-P, Heaman M, McDonald S, Lasiuk G, Sword W, et al. Barriers and facilitators of mental health screening in pregnancy. J Affect Disord. 2015;186:350–7. doi: 10.1016/j.jad.2015.06.029 26281038

[49] Nithianandan N, Gibson-Helm M, McBride J, Binny A, Gray KM, East C, et al. Factors affecting implementation of perinatal mental health screening in women of refugee background. Implement Sci. 2016;11(1):150. doi: 10.1186/s13012-016-0515-2 27863498 PMC5116191

[50] Skoog M, Berggren V, Hallström IK. “Happy that someone cared’-Non-native-speaking immigrant mothers” experiences of participating in screening for postpartum depression in the Swedish child health services. J Child Health Care. 2019;23(1):118–30. doi: 10.1177/1367493518778387 29804463 PMC7324125

[51] Soldati E, Verschuuren AE, Postma I, Veling W, Stekelenburg J, Feijen-de Jong E. Asylum-seeking women’s perspectives regarding a mental health screening method during pregnancy: a qualitative study. Trop Med Int Health. 2021;26:238–9.

[52] Hsieh WJ, Sbrilli MD, Huang WD, Hoang TM, Meline B, Laurent HK, et al. Patients’ perceptions of perinatal depression screening: a qualitative study: Study examines perinatal depression screening. Health Affairs. 2021;40(10):1612–7. doi: 10.1377/hlthaff.2021.00712 34606357

[53] Mosler F, Packer K, Jerome L, Bird V. Structured communication methods for mental health consultations in primary care: a scoping review. BMC Prim Care. 2023;24(1):175. doi: 10.1186/s12875-023-02129-y 37661251 PMC10476363

[54] Miteva D, Georgiadis F, McBroom L, Noboa V, Quednow BB, Seifritz E, et al. Impact of language proficiency on mental health service use, treatment and outcomes: “Lost in Translation”. Compr Psychiatry. 2022;114:152299. doi: 10.1016/j.comppsych.2022.152299 35220037

[55] Karliner LS, Jacobs EA, Chen AH, Mutha S. Do professional interpreters improve clinical care for patients with limited English proficiency? A systematic review of the literature. Health Serv Res. 2007;42(2):727–54. doi: 10.1111/j.1475-6773.2006.00629.x 17362215 PMC1955368

[56] Semyonov-Tal K, Lewin-Epstein N. The importance of combining open-ended and closed-ended questions when conducting patient satisfaction surveys in hospitals. Health Policy Open. 2021;2:100033. doi: 10.1016/j.hpopen.2021.100033 37383512 PMC10297822

[57] Friborg O, Rosenvinge JH. A comparison of open-ended and closed questions in the prediction of mental health. Qual Quant. 2011;47(3):1397–411. doi: 10.1007/s11135-011-9597-8

[58] Maternal Mental Health Alliance. About the Maternal Mental Health Alliance [Internet]. 2023 [cited 2026 Jan 14]. Available from: https://maternalmentalhealthalliance.org/about-us/

