# Supplementary material for: Understanding experiences and perceptions of perinatal mental health screening tools among under-served groups: A qualitative study of women from ethnic minority communities in the United Kingdom
Source: PLoS One. 2026 Apr 1;21(4):e0345882. doi: 10.1371/journal.pone.0345882 (PMC13042707; doi:10.1371/journal.pone.0345882)
Supplement: S1 Appendix — (PDF) [file pone.0345882.s001.pdf]

S1 Appendix.

EPDS	GAD-7	The Whooley questions
The EPDS was developed to assist health professionals detect new mothers suffering postpartum Depression	The GAD-7 is used for screening and measuring generalised anxiety disorders.	The Whooley questions assess women's emotional state.
The scale consists of 10 short statements, and mothers check off one of four possible answers that is closest to how they felt in the past week.	The scale consists of one question with seven short statements, and a mother checks off one of four possible answers that is closest to how she felt in the last two weeks.	The Whooley questions consist of two questions.
Postpartum depression statements from the EPDS included for example: 'have blamed myself unnecessarily when things went wrong'. Expected response included: "Yes, most of the time", "Yes, some of the time", "Not very often" and "No, never". Responses are scored 0, 1, 2, and 3 based on the severity of the symptom.	Example of statements on GAD-7 included for example: "Feeling nervous, anxious, or edge", "Not being able to stop or control worrying", "Worrying too much about different things". Expected response included: "Not at all", "Several days", "More than half the days", and "Nearly every day". Responses are scored 0, 1, 2, and 3 based on	The Whooley questions included: (1) "During the past month, have you often been bothered by feeling down, depressed, or hopeless?" And (2) "During the past month, have you often been bothered by little interest or pleasure in doing things?".

<table>
<tr><td></td><td>the seriousness of the symptom</td><td></td></tr>
<tr><td colspan="3">For the purpose of this study, women’s perceptions of perinatal and postnatal screening tools were explored by asking the study participants to comment on the Whooley questions, GAD-7, and the EPDS in terms of understanding and sensitivity of words on the scales and if they felt comfortable answering the questions truthfully. These tools were selected based on feedback from the Patient and Public Involvement and Engagement (PPIE) team, who identified them as commonly used by healthcare professionals in Sandwell.</td></tr>
</table>
