# Supplementary material for: Understanding experiences and perceptions of perinatal mental health screening tools among under-served groups: A qualitative study of women from ethnic minority communities in the United Kingdom
Source: PLoS One. 2026 Apr 1;21(4):e0345882. doi: 10.1371/journal.pone.0345882 (PMC13042707; doi:10.1371/journal.pone.0345882)
Supplement: S2 Appendix — (PDF) [file pone.0345882.s002.pdf]

Interview/focus group topic guide

This is the preliminary topic guide. The overarching objectives will remain the same, but questions and prompts will be developed as interviews/focus group discussions are undertaken to incorporate any important themes that emerge.

Date of interview	……………………………………………………………………………………
Venue of interview	……………………………………………………………………………………
(in person, online, or telephone)	……………………………………………………………………………
Pseudonym for participant	……………………………………………………………………………………

Instructions:

• Explain Council's policy of how the data will be managed [used only for this research and will not be shared outside of Council team and University of Birmingham assisting with the study – relationship subject to data sharing agreement and confidentiality clauses].

• Ensure participant reads PIS and signed consent form.

• Restate the purpose of the Interview – explore perspective/experiences of prenatal/postpartum mental health screening tools.

• Explain that you are there to understand more about their experiences and views of the effectiveness of the prenatal/postpartum mental health screening tools and that you would also talk about any other issues that are important to them that may not have been covered by the questions in this guide.

• Show participants a copy of the Whooley questions and the 10-item Edinburgh Postnatal Depression Scale (EPDS) to ensure they recall the experience.

• Check if participants have any questions.

•Start audio-recording.

•Begin the interview.

My name is Jane Hemuka and I'm the researcher working on the Maternal Mental Health Screening Tools research. Thank you for taking part in this interview. Can you just confirm for me that you have read the Information Sheet and signed the Consent Form? During the

interview, we'll be talking about your experiences during your antenatal and/or postanal mental health screening by a health professional. Shall we begin?

TOPICS TO BE COVERED IN THE INTERVIEW

1. Could you please describe your experience of being screened for emotional and mental health problems (such as depression and anxiety disorders) during and after pregnancy, using one of these (show interviewees the screening tools) mental health screening tools?

2. Did you feel comfortable answering the questions from the screening tools? Yes or No, and why?

3. Did you understand the questions from the screening tools? Yes or No, and why do you think so?

4. Do you understand the terminology/wordings used in the perinatal mental health screening tools? Yes or No, and could you explain the reason for your answer?

5. What do you think worked well during the screening program/session?

6. What do you think didn't work well during the screening program/session?

7. Were you informed of the name of the screening tool that was used? Yes or No. If Yes, what mental health screening tool was used during your screening session?

8. Were you informed of the purpose of the perinatal mental health screening tool and process that was used? Yes or No. If yes, could you please explain your experience of how the midwife discussed the purpose of the screening tool and process when you were at your first antenatal/postnatal mental health screening visit?

9. Were you informed of the result of the perinatal mental health screening? Yes or No, and how did you feel about that?

10. If you answered Yes to question 9, could you please explain your experience of how the health care professional (for example, midwife, health visitor, nurse, doctor) discussed the results of your screening when you were at your first antenatal/ postnatal mental health screening visit?

11. Do you think the screening tool used during and after your pregnancy is effective in identifying mental health problems (such as depression and anxiety disorders) amongst women from non-English ethnic groups? And why do you think so?

12. Did you answer honestly to the question from the screening tool? Yes or No, and why?

13. What do you think are the barriers that hinder the effective use of the mental health screening tools for expectant and new mothers from non-English speaking communities?

14. What do you think could improve the effective use of the mental health screening tools for expectant and new mothers from non-English speaking communities?

15. Do you think it would be beneficial for expectant and new mothers from non-English communities to take part in a mental health screening during and after pregnancy?

16. Did the midwife refer you to another service after administering the perinatal/postnatal mental health screening tools?

17. Do you have any other comments, doubts, or concerns to make about the screening tool we haven’t talked about?

This is the end of the interview/focus group. Thank you for taking part.
